# P-2188. "CMV Seropositivity is Independently Associated With Worse Lung Function in NHANES III"

**DOI:** 10.1093/ofid/ofaf695.2351

**Published:** 2026-01-11

**Authors:** Lyubov Tiegs, Madison Okuno, Jamie Forschmiedt, Ken Kunisaki, Alexa A Pragman, Kyle Rudser, Chris Wendt, David MacDonald

**Affiliations:** University of Minnesota, Saint Paul, MN; Univeristy of Minnesota, Minneapolis, Minnesota; University of Minnesota, Saint Paul, MN; Boise VA Medical Center, Boise, Idaho; Minneapolis VA Medical Center and University of Minnesota, Minneapolis, Minnesota; University of Minnesota, Saint Paul, MN; Minneapolis VA Medical Center, Minneapolis, Minnesota; University of Minnesota, Saint Paul, MN

## Abstract

**Background:**

Chronic obstructive pulmonary disease (COPD) is the 4^th^ leading cause of death globally. COPD is defined by irreversible expiratory airflow obstruction (e.g., FEV1/FVC< 0.70) and severity is assessed by reduced FEV1. COPD is most often caused by inhalation of tobacco smoke, but even after controlling for smoking and other known risk factors, much of the risk remains unexplained. Latent cytomegalovirus (CMV) has been hypothesized to affect lung function through effects on natural killer cells, systemic inflammation, and direct effects on lung tissue. Prior analyses have been performed in populations with limited racial/ethnic, biologic sex, and/or geographic representation. The National Health and Nutritional Examination Survey (NHANES III) was a population-based sample from the United States.

**Table 1:**
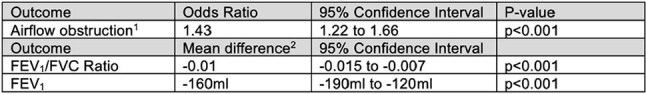
Associations between CMV seropositivity and lung function. All analyses were adjusted for age, race/ethnicity, biologic sex, smoking, poverty-income ratio, education, and urban vs rural residence. FEV1, forced expiratory volume in 1-second; FVC, forced vital capacity 1: Defined as FEV1/FVC ratio < 0.7. 2: Mean differences represent differences in the outcome of interest (ratio for FEV1/FVC ratio and liters for FEV1) in participants positive for CMV compared to those negative for CMV.

**Table 2:**
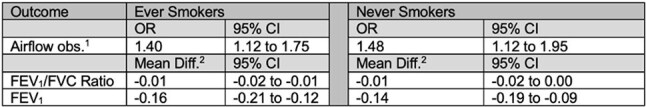
Associations between CMV seropositivity and lung function in ever smokers and never smokers. All analyses were adjusted for age, race/ethnicity, biologic sex, smoking, poverty-income ratio, education, and urban vs rural residence. All interaction p-values (not shown) were greater than 0.4. CI, confidence interval; OR, odds ratio. 1: Defined as FEV1/FVC ratio < 0.7. 2: Mean differences represent differences in the outcome of interest (ratio for FEV1/FVC ratio and liters for FEV1) in participants positive for CMV compared to those negative for CMV.

**Methods:**

Using cross-sectional data from NHANES III, we tested associations between CMV seropositivity (ELISA optical density index values greater than 1.0) and the following outcomes: 1) airflow obstruction [FEV_1_/forced vital capacity (FVC) < 0.7]; 2) FEV_1_/FVC (continuous), and FEV_1_ (continuous). Binary outcomes were analyzed using logistic regression and continuous outcomes were analyzed using linear regression. All analyses used robust variance estimation for confidence intervals and P-values and were adjusted for important covariates listed in the tables below.

**Results:**

Among 14,279 participants with available spirometry and CMV IgG serologies, 11,000 were CMV positive and 3,279 were CMV negative. The mean age of included participants was 42.5 (SD 16.6) years, 51.2% were female, and 54.2% were ever smokers. CMV seropositivity was associated with higher odds of airflow obstruction and lower FEV_1_/FVC ratio and FEV_1_ (Table 1). Results did not significantly differ in never smokers vs ever smokers (Table 2).

**Conclusion:**

In a general population sample of the United States, CMV seropositivity was independently associated with worse lung function. If these results are confirmed in longitudinal studies, CMV vaccines and other CMV mitigation efforts could have a role in reducing the burden of COPD.

**Disclosures:**

Ken Kunisaki, MD, MS, Nuvaira: Data and Safety Monitoring Board Member

